# 完整糖基化肽段的富集与质谱解析新技术研究进展

**DOI:** 10.3724/SP.J.1123.2021.06011

**Published:** 2021-10-08

**Authors:** Luyao LIU, Hongqiang QIN, Mingliang YE

**Affiliations:** 1.中国科学院大连化学物理研究所, 中国科学院分离分析化学重点实验室, 辽宁 大连 116023; 1. CAS Key Laboratory of Separation Science for Analytical Chemistry, Dalian Institute of Chemical Physics, Chinese Academy of Sciences, Dalian 116023, China; 2.中国科学院大学, 北京 100049; 2. University of Chinese Academy of Sciences, Beijing 100049, China

**Keywords:** 蛋白质糖基化, 完整糖肽富集, 质谱分析, 谱图解析, protein glycosylation, intact glycopeptide enrichment, mass spectrometric analysis, spectral interpretation

## Abstract

蛋白质糖基化是生物体内最重要的翻译后修饰之一,在蛋白质稳定性、细胞内和细胞间信号转导、激素活化或失活和免疫调节等生理过程和病理进程中发挥重要作用。而异常的蛋白质糖基化往往和多种疾病的发生发展密切相关,目前应用于临床检测的多种肿瘤生物标志物大多属于糖蛋白或者糖抗原。因此在组学层次系统分析蛋白质糖基化的变化对阐明生物体内糖基化修饰的调控机理和发现新型疾病标志物都非常重要。基于质谱的蛋白质组学技术为全面分析蛋白质及其修饰提供了有效的分析手段。在自下而上的蛋白质组学研究中,由于完整糖基化肽段同时存在性质各异的肽段骨架和糖链结构、糖肽的相对丰度和离子化效率较低以及糖基化修饰有高度异质性等特点,完整糖肽的分析比其他翻译后修饰更加困难。近年来,为了更全面、系统地分析蛋白质糖基化,研究人员发展了一些新技术,包括完整糖肽的富集技术、质谱的碎裂模式和数据采集模式、质谱数据的解析方法和定量策略等等,大力推进了该领域的研究水平,也为研究蛋白质糖基化相关的生物标志物提供了技术支持。该篇综述主要关注近年来基于质谱的糖蛋白质组学研究中的新进展,重点介绍针对完整*N*-和*O*-糖基化肽段的富集新技术和谱图解析新方法,并讨论其在肿瘤早期诊断方面的应用潜力。

## Abstract (en)

Protein glycosylation is one of the most important post-translational modifications (PTMs). The glycosylation is crucial in a variety of physiological and pathological processes that include protein stability, intracellular and intercellular signal transduction, hormone activation or inactivation, and immune regulation. Protein glycosylation is generated by complex biosynthetic pathways comprising hundreds of glycosyltransferases, glycosidases, transcriptional factors, transporters, and protein backbones. Abnormal protein glycosylation is closely associated with the occurrence and development of diseases. Many disease biomarkers in clinical screening are glycoproteins (alfa fetoprotein for liver cancer, carbohydrate antigen 125 for ovarian cancer, carcinoembryonic antigen for colon cancer, prostate-specific antigen for prostate cancer, etc.), and glycan antigens (carbohydrate antigen 19-9 for gastrointestinal cancer and pancreatic cancer, etc.). Glycoproteomics research and technological developments are important to elucidate the mechanism of protein glycosylation in vivo. Mass spectrometry (MS)-based proteomics provides an excellent approach for the comprehensive analysis of proteins and their modifications. In bottom-up proteomics, glycoproteomic analysis is more difficult than other PTMs because intact glycopeptides have diverse peptide backbones and glycan chains, relatively low abundance and ionization efficiency, and pronounced heterogeneity. In recent years, glycoproteomic methodologies such as intact glycopeptide enrichment methods, MS fragmentation and acquisition approaches, MS data interpretation tools and software, and quantification strategies have been appreciably improved. These methodologies have driven in-depth glycoproteomics research. This review focuses on the recent advances in MS-based glycoproteomics. New enrichment methods and spectral interpretation approaches of intact *N*- and *O*-glycopeptides are discussed. Their applications in answering various questions in complex biological systems are also considered.

The new enrichment methods for intact glycopeptides are mostly based on existing principles. Some properties of the materials, such as hydrophilicity and electrophilicity, have been optimized to improve the enrichment performance. For example, dual-functional Ti(IV)-IMAC materials have been used for the separation of glycopeptides and phosphopeptides. Considering the clinical applications, some glycoproteomics methods integrate enrichment processing into automated workflows to reduce errors caused by manual operations and to increase the experimental reproducibility and efficiency. For example, an automated glycopeptide enrichment method consisting of a liquid chromatograph equipped with a hydrophilic interaction chromatography column has been shown capable of highly reproducible analyses of site-specific glycopeptides in complex biological samples. These methods are more suitable for the discovery of newly glycosylation-related biomarkers as well as for the physiopathological studies of human diseases.

With the optimization of glycopeptide enrichment methods and the innovation of MS technologies in the past decade, MS analysis of intact glycopeptides has begun to yield a wealth of glycopeptide fragment ions and plentiful high-quality MS data. This review introduces several effective fragmentation methods for intact glycopeptides. These include collision-induced dissociation, high-energy collision dissociation, electron capture dissociation, electron-transfer dissociation, and electron-transfer/higher-energy collision dissociation. Automated analysis of MS data of intact *N*- and *O*-glycopeptides requires interpretation approaches and corresponding software tools with high sensitivity and reliability. Finally, we highlight the utility of several spectral interpretation approaches and their corresponding popular search software, including ArMone, Byonic, GPQuest, pGlyco, O-search, MSFragger-Glyco, and O-Pair Search. In addition, MS data acquisition modes, such as data-dependent acquisition, data-independent acquisition, multiple reaction monitoring technology, and parallel reaction monitoring technology, have great application prospects in glycoproteomics research. With the improvements in enrichment methods, MS technologies, and spectral interpretation approaches for intact *N*- and *O*-glycopeptides, comprehensive and systematic glycoproteomics analysis has tremendously expanded the knowledge of protein glycosylation. These glycoproteomic technologies have a wide range of applications that include exploring the molecular mechanisms of protein glycosylation and discovering the new biomarkers of human diseases.

蛋白质糖基化是一种非常普遍、复杂和多样的蛋白质翻译后修饰。在真核生物中,蛋白质糖基化过程主要发生在内质网和高尔基体系统内,有大约200个糖基转移酶参与,并使得50%~70%的蛋白质被糖基化修饰^[1]^。根据糖链和蛋白质连接方式的不同,蛋白质糖基化主要分为*N*-糖基化、*O*-糖基化、*C*-糖基化和糖基磷脂酰肌醇(GPI)锚定4大类型,其中对*N*-和*O*-糖基化的研究最为广泛。在*N*-糖基化中,糖链通过*N*-乙酰葡萄糖胺(GlcNAc)与天冬酰胺(Asn)侧链氨基共价结合,其糖链有五糖核心结构,糖基化位点具有特定肽段序列Asn-X-Ser/Thr(X≠Pro)。在*O*-糖基化中,糖链通过与丝氨酸(Ser)或苏氨酸(Thr)侧链羟基共价结合,其糖链没有固定的核心结构,糖基化位点也没有保守肽段序列。*N*-和*O*-糖基化在蛋白质稳定性、细胞内和细胞间信号转导、激素活化或失活和免疫调节等方面起着重要作用^[2]^。蛋白质糖基化的异常表达也通常预示着相关疾病的发生发展,比如炎症、癌症、遗传病等。目前,大多数被批准临床使用的肿瘤生物标志物是糖蛋白(例如用于肝癌的甲胎蛋白(AFP)、用于卵巢癌的癌抗原125(CA125)、用于结肠癌的癌胚抗原(CEA)、用于前列腺癌的前列腺特异抗原(PSA)等)或糖抗原(例如用于胃肠癌和胰腺癌的癌抗原19-9(CA19-9)等)^[3]^。因此,糖蛋白质组学分析对于疾病的早期筛查和病理研究等非常重要。

基于质谱的蛋白质组学技术为全面分析蛋白质及其修饰提供了极好的分析手段。在自下而上的蛋白质组学研究中,单一蛋白质或者复杂生物样品(细胞、组织、体液等)的全蛋白通常经酶解成肽段后再进行高效液相色谱-串联质谱(HPLC-MS/MS)的鉴定和定量分析。然而,由于糖链的多样性和高度异质性,全面分析蛋白质糖基化仍具有很大的挑战。一方面,尽管蛋白质糖基化普遍存在,但是糖蛋白相对于全蛋白的丰度较低,而完整糖肽的含量更低,难以进行质谱的直接检测。因此,为了提高复杂生物样品中完整糖肽的丰度,发展完整糖肽的富集方法具有重要意义。目前,常见的糖肽富集技术有亲水作用色谱法、金属亲和色谱法、凝集素亲和色谱法、硼酸化学法、酶化学法、酰肼化学法等等。另一方面,相比于非修饰肽段,糖肽的低离子化效率和糖链的高度异质性使得完整糖肽的质谱表征具有挑战性。因此,开发有效的质谱碎裂方法和谱图解析工具对完整糖肽的综合分析非常重要。近10年以来,随着质谱技术的革新,谱图数据的质量越来越高,用于精确解析完整*N*-和*O*-糖肽的软件算法也得到了飞速的发展^[4]^。

在本篇综述中,我们重点关注用于糖蛋白质组学分析的糖肽富集技术和谱图解析方法的最新进展:首先介绍几种不同机理的完整糖肽富集方法,并重点介绍这些方法最新的技术进展及其应用;然后介绍近年来发展的完整糖肽质谱碎裂方法,并重点介绍完整糖肽的谱图解析策略和对应的数据分析软件;并对蛋白质糖基化研究的发展方向进行了展望。

## 1 完整糖肽富集方法的新进展

基于质谱的糖蛋白质组学技术是分析位点特异性蛋白质糖基化的有效手段。为了提高质谱分析的灵敏度,从复杂生物样本的全蛋白酶解样品中特异性富集完整糖肽的步骤是必要的^[5,6]^。近年来,研究人员通过开发和改进各种富集方法,极大地提高了不同样本中完整糖肽的富集效率。

### 1.1 亲水作用色谱法

亲水作用色谱法(hydrophilic interaction liquid chromatography, HILIC)是一种广谱的富集方法,其通过糖基化肽段和非糖基化肽段之间极性的差异实现完整糖肽的分离和富集。与其他富集方法相比,HILIC的优势是既能保留完整糖肽的结构,也能无偏差地富集所有类型的完整糖肽。但是HILIC的缺点之一是糖肽和非糖肽的共流出导致富集特异性较低,这需要开发具有更强亲水基团的HILIC材料来提高富集效率。目前,应用广泛的HILIC材料有两性离子功能化材料(如ZIC-HILIC)、碳水化合物功能化材料(如Click Maltose-HILIC)、酰胺功能化材料和基于金属有机框架的亲水材料(metal-organic frameworks, MOF)等^[7,8,9]^。

由于*N*-和*O*-糖基化的异质性高,通过糖苷酶(例如PNGase F糖苷内切酶)释放*N*-糖链可以简化*N*-糖基化的分析,但是缺少能释放*O*-糖链的糖苷酶,因此蛋白质*O*-糖基化的分析具有很大挑战。最近,You等^[10]^提出了一个结合HILIC富集和化学法去唾液酸化的策略,通过在一定程度上简化*O*-糖链的方式,提高了鉴定*O*-GalNAc糖基化的灵敏度。他们将该方法进一步应用于人血清样本的分析,较高可信度地鉴定到来自94个糖蛋白的185个*O*-GalNAc肽段序列。为了深度研究疾病中蛋白质糖基化的异常,通常需要对大量生物样本进行检测。因此,在开发高特异性的富集材料的同时也需要发展高重现性、高稳定性和易操作性的完整糖肽富集平台,这对于支持规模化的糖蛋白质组学研究非常关键。近期,我们^[11]^发展了一种自动化的完整糖肽富集方法,该方法通过配置Click Maltose-HILIC微柱的液相色谱仪实现了对微量起始血清酶解液中完整*N*-糖基化肽段的富集(见图1)。该方法能从1 μL起始血清中富集并鉴定到近1200条完整*N*-糖肽,同时其定量分析的重现性高,长期运行的稳定性好,适合大队列样本的糖蛋白质组学研究。我们进一步将该方法应用于4例胰腺癌患者血清中位点特异性*N*-糖基化的分析,发现患者组IgG1蛋白180号*N*-糖基化位点上单岩藻糖无唾液酸糖型的表达量显著低于健康对照组。

**图1:**
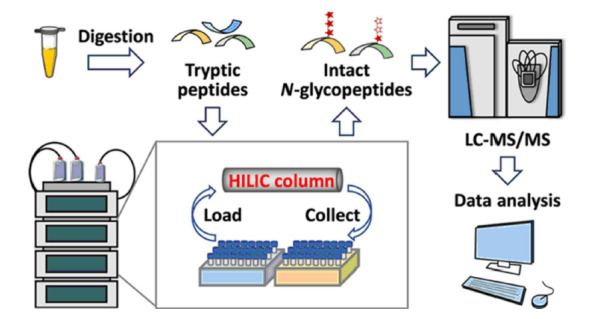
基于亲水作用色谱法的自动化完整糖肽富集平台^[11]^

### 1.2 金属亲和色谱法

金属氧化物亲和色谱法(metal oxide affinity chromatography, MOAC)和固定金属离子亲和色谱法(immobilized metal ion affinity chromatography, IMAC)是两种最典型的金属亲和色谱法,它们最早被广泛地应用于蛋白质磷酸化肽段的富集;由于糖基化肽段和磷酸化肽段都含有丰富的负电荷修饰基团,并且有相似的强亲水性,金属亲和色谱法也逐渐应用于蛋白质糖基化肽段的富集。

MOAC通过金属氧化物与修饰肽段之间的配位相互作用实现糖肽的选择性富集。二氧化钛(TiO_2_)的稳定性高和非特异性结合少,是MOAC中最具代表性的富集材料,能够选择性地富集带唾液酸的完整糖肽。为了提高TiO_2_对糖肽的富集能力,段梅等^[12]^尝试了将TiO_2_介孔气凝胶材料应用于糖肽的选择性富集,发现其富集性能和选择性均显著优于商品化纳米级的TiO_2_颗粒。为了同时结合MOAC和HILIC的富集优势,Sun等^[13]^制备了一种双功能磁性纳米材料(亚氨基二乙酸修饰的磁性二氧化钛,Fe_3_O_4_@TiO_2_-IDA),实现了对复杂生物样本中磷酸化肽段和糖基化肽段的分离富集,并从100 μg小鼠大脑样品的单针LC-MS/MS分析中同时鉴定到550条磷酸肽和330条完整*N*-糖肽。

IMAC以过渡态金属阳离子(如Fe^3+^、Ga^3+^、Zr^4+^等)作为亲和基团,与带负电荷的修饰肽段之间发生静电相互作用实现糖肽的选择性富集。甘露糖-6-磷酸(M6P)糖基化是一种重要的翻译后修饰,在将溶酶体水解酶转移到溶酶体中起着至关重要的作用,而异常M6P修饰与溶酶体储存疾病(包括阿尔茨海默病和癌症)有关。但是M6P修饰的丰度极低,这使得完整M6P糖肽的研究仍然具有挑战性,因此开发完整M6P糖肽的富集方法尤为重要。Huang等^[14]^首先发现螯合了Ti^4+^的IMAC(Ti(IV)-IMAC)材料包含大量的磷酸螯合Ti(IV)离子,能通过静电相互作用富集磷酸肽;同时该材料含有大量高度亲水的羟基、氨基和磷酸根基团,能通过亲水相互作用富集糖肽。因而,他们发现该双功能Ti(IV)-IMAC材料能有效地富集M6P糖蛋白,并从小鼠5种组织(肝、心、肺、肾、脑)中分析鉴定到来自81个M6P糖蛋白的237个完整M6P糖肽,第一次实现了小鼠样本中M6P糖基化的大规模分析。

随后根据不同修饰肽段之间静电特性和亲水性的差异,Huang等^[15]^进一步通过调控洗脱条件,利用该双功能Ti(Ⅳ)-IMAC材料实现了中性糖肽和唾液酸糖肽、单磷酸肽和多磷酸肽的分离,进而显著提高了糖肽、磷酸肽和完整M6P糖肽的覆盖率和鉴定量(见图2a)。近期,Yue等^[16]^发现Ti(IV)-IMAC材料也能富集复杂生物样本中的完整*O*-糖肽,并且能从0.1 μL人血清中富集约200条完整*O*-GalNAc糖肽,他们将该方法应用于研究人肝癌血清样本中蛋白质*O*-糖基化的异常变化(见图2b)。

**图2:**
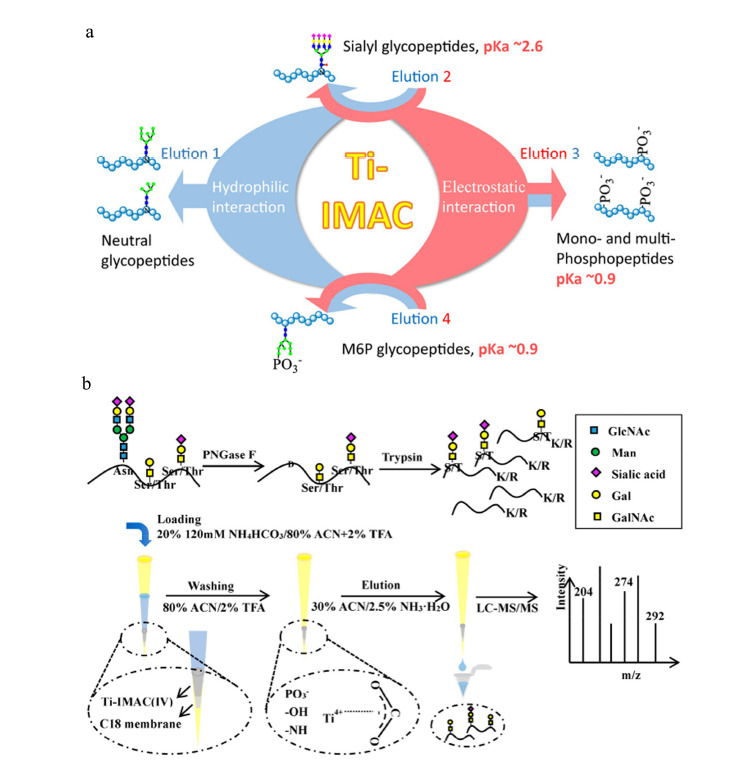
双功能Ti(IV)-IMAC材料^[15,16]^

### 1.3 凝集素亲和色谱法

凝集素亲和色谱法(lectin affinity chromatography, LAC)通过固载在琼脂糖或者磁珠上的凝集素选择性地识别有特定结构的单糖或聚糖实现完整糖肽的分离和富集。根据凝集素的特异性,该方法被广泛地用于特定*N*-和*O*-糖蛋白和糖肽的富集。最常用于富集*N*-糖基化肽段的凝集素有:刀豆蛋白A凝集素(concanavaline A, Con A)能特异性结合α-甘露糖;小麦胚芽凝集素(wheat germ agglutinin, WGA)能特异性结合GlcNAc和唾液酸。常用于富集*O*-GalNAc型糖基化肽段的凝集素有:木菠萝凝集素(Jacalin)能特异性识别半乳糖基(*β*-1,3) *N*-乙酰半乳糖胺(Gal(*β*-1,3)GalNAc);野豌豆凝集素(VVA)能特异性识别*α*-或*β*-末端*N*-乙酰半乳糖胺(GalNAc)。单凝集素亲和色谱法通常应用于复杂的生物样品(如血清、血浆和组织裂解液等),以监测疾病中特定蛋白质糖基化的变化。Tsai等^[17]^建立了一种结合WGA富集方法和多重反应监测(MRM)质谱技术的检测方法,通过鉴定结直肠癌患者血浆中经WGA捕获的糖蛋白和对应多肽表达量的变化,从而监测早期结直肠癌的发生。相比于单凝集素亲和色谱法,多凝集素亲和色谱法通过混合多种凝集素因而能同时富集更多样化的蛋白质糖基化。Gbormittah等^[18]^利用组合了Con A、WGA和Jacalin多凝集素亲和色谱法的富集平台,成功地富集和表征了胰腺囊肿液中不同水平的糖蛋白,并用于探索差异表达蛋白来区分黏液性和非黏液性囊肿液。

### 1.4 硼酸化学法

硼酸化学法利用硼酸与单糖形成可逆共价键实现完整糖肽的富集。具体原理是在碱性条件中,硼酸基团与糖链的顺式二醇结构反应形成环状的硼酸酯,从而捕获完整糖肽;在酸性条件下,硼酸酯能可逆水解,从而释放完整糖肽并且不影响糖链的结构。由于糖链有多羟基的结构特点,硼酸化学法在富集完整糖肽方面具有很大潜力。然而,硼酸和不同糖链之间的亲和力不同,限制了该方法的普适性。2018年,Xiao等^[19]^开发了一种硼酸化合物偶联树枝状聚合物用于促进低丰度糖肽的富集,通过比较不同硼酸衍生物的富集性能,发现苯并溴唑能显著增强复杂样品糖肽的覆盖度。他们利用该方法从人类细胞(MCF7、HEK 293T和Jurkat)中富集并鉴定到来自1906个蛋白质的4691个*N*-糖基化位点。最后他们发现尽管*O*-GlcNAc不含顺式二醇,但是该方法也能高效地从人类细胞中富集超过200个*O*-GlcNAc蛋白。近期,Chen等^[20]^组合使用硼酸化学法和HILIC两种方法富集人脑脊液(cerebrospinal fluids, CSF)样本中的完整*N*-糖肽,共鉴定到来自285个蛋白质的511个糖基化位点和2893条完整*N*-糖肽,得到了目前最大的位点特异性*N*-糖蛋白质组学数据,他们进一步将该方法应用于深入分析阿尔茨海默病CSF样本的*N*-糖蛋白质组学。

### 1.5 酶化学法

酶化学法同时结合了酶反应和化学反应实现糖基化肽段的识别、捕获和释放,是糖蛋白质组常用的研究方法之一。Zheng等^[21]^设计了一种酶化学法用于富集含Tn抗原(*O*-GalNAc)的糖蛋白。该方法的原理是利用半乳糖氧化酶将GalNAc上的羟基氧化成醛基,随后利用酰肼化学捕获上述糖肽,最后利用甲氧胺释放糖肽,实现含Tn抗原糖蛋白的分离和富集。他们利用该方法从Jurkat细胞中鉴定到96个含Tn抗原的糖蛋白。随后,You等^[22]^发展了一种用于富集黏蛋白型核心1结构*O*-糖肽(mucin-type core-1 *O*-糖肽,也称为*O*-GalNAc糖肽)的酶化学法。在去除了*N*-糖链的血清酶解液样品中,该方法首先去除糖链的唾液酸,使Gal/GalNAc残基暴露在糖链末端,随后利用半乳糖氧化酶氧化羟基成醛基,酰肼化学法共价捕获醛基,最后使用羟胺及其同系物释放糖肽。最终他们使用该方法从50 μL人血清中鉴定到来自38个糖蛋白的59条*O*-GalNAc修饰的肽段序列,展现了其在复杂生物样本*O*-糖基化蛋白质组中研究的潜力。2018年,Yang等^[23]^报道了一种新型的位点特异性提取*O*-糖肽的方法(简称为EXoO),用于黏蛋白型*O*-糖基化的大规模分析。他们首先将多肽的N端经还原氨化共价结合至一个固定载体,随后使用一种特异性蛋白酶(OpeRATOR酶)酶切*O*-糖基化位点Ser/Thr的N端从而释放*O*-糖肽,实现*O*-糖肽的选择性富集。最终,他们利用该方法从人肾脏、T细胞和血清3种样本中共鉴定到来自1000多个糖蛋白的超过3000个*O*-糖基化位点。

### 1.6 其他方法

酰肼化学法首先氧化糖链上的顺式邻二羟基生成醛基,随后醛基与酰肼树脂上的氨基不可逆反应形成共价的腙键,实现糖肽的捕获;最后*N*-糖链通过PNGase F酶或者化学法去除,实现富集肽段的释放。在早期受限于质谱检测技术,质谱的碎裂模式和采集速度不适合分析完整糖肽,而酰肼化学法通过简化蛋白质糖基化,在肽段层次有效定位了*N*-糖基化位点,因而得到了广泛的应用。近年来,随着质谱仪的发展和谱图解析软件的开发,实现了对完整糖肽的高可信度表征。酰肼化学法的实验流程复杂,尤其是丢失了糖链的信息,使其难以应用于位点特异性糖型的研究。

多孔石墨碳(porous graphitized carbon, PGC)是一种新型的碳材料^[24]^,其通过保留传统反相中不能保留的高极性化合物而实现糖肽的富集。但是目前PGC对完整糖肽的保留机制(偶极、亲水性、电荷诱导、分散等)并不是很清楚。Xue等^[25]^系统地研究和比较了硼酸化学、ZIC-HILIC和PGC在富集完整*N*-糖肽方面的性能,发现硼酸化学倾向于捕获高甘露糖型糖肽,ZIC-HILIC富集得到的*N*-糖肽最多且无偏向性,而PGC不适合富集肽段序列较长的糖肽,尤其是胰蛋白酶消化肽段。

生物正交法是一种靶向标记和富集糖蛋白的新方法。该方法结合了代谢标记和点击化学,首先在细胞培养中引入含有生物正交官能团(如叠氮化物、炔烃或酮等)的非天然糖类,对细胞内糖类化合物进行代谢标记,随后通过点击化学反应将标记的糖蛋白与亲和探针共价结合,从而实现糖蛋白的捕获和富集。Hubbard等^[26]^将该方法应用于富集细胞表面糖蛋白,并从前列腺癌细胞株中鉴定到超过70种细胞表面糖蛋白。Spiciarich等^[27]^进一步将该方法应用于体外培养的人体组织,他们分别对正常和癌变的前列腺组织进行切片和培养,并从前列腺癌组织中鉴定出一些高表达的糖蛋白,拓展了该方法的临床应用。目前该方法广泛应用于糖蛋白层次的富集,在位点特异性蛋白质糖基化的鉴定上仍有很大的发展空间。

## 2 质谱鉴定和谱图解析方法的新进展

在自下而上的糖蛋白质组学中,完整糖肽经分离和富集后进入高分辨质谱仪,得到含丰富碎片离子的谱图,随后通过谱图解析技术实现对完整糖肽和糖蛋白的表征^[28]^。基于质谱的糖蛋白质组学方法具有高灵敏度和高质量精度等优点,但是对位点特异性蛋白质糖基化的鉴定和定量依赖于有效的质谱分析方法和谱图解析策略的发展。如今,这两项重大突破显著促进了位点特异性完整糖肽的表征,分别是:用于获得丰富糖链和肽段碎片信息的质谱解离方法的发展;用于解析完整糖肽质谱谱图的检索引擎的开发^[29]^。

### 2.1 完整糖肽的质谱碎裂方法

复杂生物样品中的完整糖肽经过分离和富集后,进入生物质谱进行糖蛋白质组学分析。不同类型的生物质谱有不同的电离技术、碎裂方式和数据采集模式等,在糖蛋白质组学分析中具有各自的特点与优势。目前用于糖蛋白质组学分析的质谱电离技术主要有基质辅助激光解析电离(matrix-assisted laser desorption/ionization, MALDI)和电喷雾电离(electrospray ionization, ESI)这两种代表性的软电离技术。MALDI-MS是一个能有效地分析*N*-糖链的质谱技术,其操作简单快速,并且能分析极微量的样品,这对珍贵的临床样本来说非常重要。由于肿瘤的发生发展伴随着异常的糖基化修饰,这种异常糖基化一定程度表现在糖蛋白糖链丰度的变化,因此利用MALDI-MS能有效地对糖链变化进行检测。Ren等^[30]^提出了一个用于肿瘤筛查的糖链生物标志物,称为IgG半乳糖比值(IgG Gal-ratio)。他们通过MALDI-MS测定血清IgG中无半乳糖基糖链(G0)、单半乳糖基糖链(G1)和双半乳糖基糖链(G2)这3种*N*-糖链的相对强度,并进一步计算G0/(G1+G2×2)的比值。他们发现该比值能够有效地区分12种癌症(胃癌、肝癌、肺癌、卵巢癌、结肠直肠癌、食道癌、胰腺癌、肾癌、前列腺癌、膀胱癌、乳腺癌和宫颈癌)和非癌症对照,作为一种用于癌症筛查的非侵袭性的生物标记物有着巨大的潜力。但是MALDI-MS也有着很大的局限性,比如其碎裂能量导致唾液酸和岩藻糖的丢失;分析糖链时不能区分糖型异构体;分析完整糖肽时不能实现糖基化位点定位等等。和MALDI-MS相比,ESI-MS在检测分析完整糖肽方面有着更广泛的应用,例如强负电性的唾液酸不能在MALDI-MS中得到良好的信号响应但能在ESI-MS中被检测到。尤其是ESI-MS前端串联液相色谱(LC-ESI-MS)能够在线分离复杂生物样本,降低了样品复杂度,提高了检测能力。进一步,液相色谱串联多级质谱(LC-MS^n^)能获得更加丰富的完整糖肽的特征碎片离子,能在蛋白质组学层次实现完整糖肽的组成和位点的鉴定,进一步增加了完整糖肽分析的深度,是目前最可靠的分离同分异构体的质谱方法。

在糖蛋白质组学质谱分析中,完整糖肽同时含有糖链和肽段,经质谱碎裂后产生碎片离子的形式更加多样化,包括肽段碎片离子(b/y离子;c/z离子)和糖碎片离子(Y离子)等。完整糖肽分析中常用的质谱碎裂方式有碰撞诱导解离(collision-induced dissociation, CID)、高能量碰撞解离(higher-energy collision dissociation, HCD)、电子捕获解离(electron capture dissociation, ECD)、电子转移解离(electron-transfer dissociation, ETD)、电子转移/高能碰撞解离(electron-transfer/higher-energy collision dissociation, EThcD)等。CID和HCD都是基于碰撞能量再分配的原理碎裂糖链和肽段,并主要形成b/y离子和Y离子。完整糖肽中糖苷键和肽键裂解所需的能量差异大,CID的碎裂能量低,主要碎裂糖苷键并产生丰富的Y离子。从CID谱图中能够有效地分析糖链的组成和结构,但是不能获得糖基化位点和肽段序列的信息,应用范围受限。和CID相比,HCD的碎裂能量更高、能量分布更均匀和激活时间更短,因此HCD能裂解完整糖肽并产生丰富的b/y离子和Y离子。研究表明,不同的HCD能量裂解完整糖肽所产生的碎片离子种类大不相同,较低HCD能量倾向于产生Y离子,而较高HCD能量倾向于产生b/y离子^[31]^。因此,与单一HCD能量相比,多级HCD能量可以产生更丰富的互补碎片离子峰,因而获得更加详细的糖肽结构信息。针对完整糖肽的肽段碎片离子少、多级碎裂中鉴定效率低的问题,Qin等^[32]^报道了一种基于酶辅助的拟多级谱策略用于*N*-完整糖肽的表征。他们通过匹配完整*N*-糖肽质谱数据和去糖基化后的肽段质谱数据之间的保留时间、相对分子质量等,确定完整糖肽的肽段序列和糖链的组成,实现完整糖肽的高效鉴定。最后他们将该策略应用于人源肝癌和癌旁组织之间*N*-糖基化的差异分析,共鉴定到4471个位点特异性*N*-糖型,是当时肝组织*N*-糖蛋白质组的最大数据集,并筛选到多个候选差异蛋白,有望提高肝癌诊断的准确性。ECD和ETD主要碎裂完整糖肽的肽段骨架并产生丰富的c/z离子,有利于糖基化位点和肽段序列的鉴定,是目前完整糖肽结构解析的强大工具之一。但是ECD和ETD裂解完整糖肽时,一方面依赖于母离子的电荷与质荷比导致碎裂效率低,另一方面能保持糖链结构导致糖链碎片离子信息的缺失,使其分析灵敏度差,应用受限。随着技术的发展,EThcD是近年来新的集成碎裂技术^[33]^,整合了HCD和ETD这两种碎裂模式的优势,能产生完整糖肽的b/y/c/z多种碎片离子,获得更加丰富的特征离子信号用于完整糖肽的结构解析。

### 2.2 完整糖肽的谱图解析方法和软件

受限于早期质谱条件,基于质谱的糖蛋白质组学研究策略依赖于对完整糖肽的糖链和肽段的分别分析。尽管这种策略能简化蛋白质糖基化的鉴定,但是缺失糖基化异质性和位点特异性等信息,不能实现完整糖肽的深入分析。随着完整糖肽富集技术的优化和质谱检测技术的革新,糖蛋白质组学分析获得了丰富的完整糖肽碎片离子,得到了高质量的质谱数据。为了对这些质谱数据进行自动、快速、高灵敏度和高可信度的解析,开发完整*N*-和*O*-糖肽的谱图解析策略和相应数据分析软件有着重要意义。

在*N*-糖基化中,*N*-糖链存在五糖核心但是其完整结构的多样性高,使得完整*N*-糖肽谱图的复杂性高,为准确地解析蛋白质*N*-糖基化数据带来了困难。相比于*N*-糖基化,*O*-糖基化的谱图解析要更加复杂,一方面*O*-糖链没有固定核心结构同时类型繁多,另一方面一条肽段上可能存在多个*O*-糖基化位点,这些为高通量分析完整*O*-糖肽带来了极大的挑战。近年来,针对完整*N*-和*O*-糖肽经质谱碎裂后获得的各种碎片离子特征,研究人员开发了一些谱图解析策略来鉴定完整糖肽,以期实现对复杂生物样本完整糖肽的大规模分析。

Cheng等^[34]^开发了一个高通量的完整*N*-糖肽分析软件平台ArMone,用于解析*N*-糖基化修饰位点以及糖链结构。通过平行的LC-MS/MS分析完整糖肽和其对应的去糖基化肽段,他们发现这两类肽段在色谱保留时间上具有一致性。因此他们首先根据去糖基化肽段HCD谱图实现肽段序列的高可信度鉴定,随后通过匹配糖数据库和含氧鎓离子的完整糖肽HCD谱图,根据*N*-糖链的五糖核心确定糖链信息,最后实现完整*N*-糖肽的鉴定。他们利用该方法从人源胚胎肾细胞系中鉴定到了2249条非冗余的糖肽序列及糖链结构,这是当时最大的完整*N*-糖肽鉴定数据集。Byonic是一个商用检索软件^[35]^,能分别检索和鉴定完整*N*-和*O*-糖肽。它通过在糖数据库中添加了人源及哺乳动物中常见的*N*-和*O*-糖型,将每个糖型视为谱图数据库搜索的可变修饰,从HCD或ETD谱图中鉴定完整糖肽。GPQuest是一种基于HCD谱图鉴定位点特异性完整*N*-糖肽的算法^[36]^。它首先采用不同的切糖链鉴定策略建立了一个肽段谱图库,随后通过匹配谱图库和完整糖肽的HCD谱图鉴定肽段序列,推断糖链质量,最后实现完整糖肽的鉴定。

pGlyco是一个精准分析*N*-糖蛋白质组学的软件^[31]^,它首先利用完整*N*-糖肽经阶梯HCD能量碎裂获得谱图,通过与糖数据库匹配以鉴定糖组成,随后通过与蛋白质组数据库匹配以鉴定肽段序列,最后从糖链、多肽和糖肽3个层面综合控制假阳性率,增强了完整*N*-糖肽鉴定的准确性(见图3)。他们利用该数据分析平台分别分析了5个小鼠组织(大脑、心脏、肝脏、肾脏、肺)并得到了一个大规模的*N*-糖蛋白质组数据集,包括了来自955个糖蛋白的1988个*N*-糖基化位点上的10009个不同的位点特异性*N*-糖链。

**图3:**
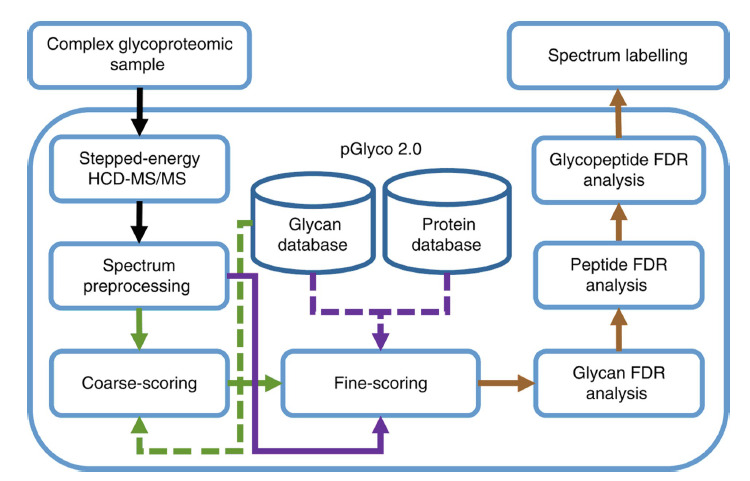
pGlyco 2.0检索策略用于解析完整*N*-糖肽HCD谱图^[31]^

Mao等^[37]^提出了一种分析完整*O*-糖肽HCD谱图的新方法O-Search,该方法不在Ser/Thr残基上设置可变修饰,而是在肽段层次检测*O*-糖基化导致的质量偏移,其分析流程主要包含:(1)通过丰度最高的两个鎓离子(HexNAc-2H_2_O-CH_2_O^+^, *m/z* 138.05; HexNAc^+^, *m/z* 204.09)过滤谱图,只保留糖肽谱图;(2)从糖肽谱图母离子中扣除可能的*O*-糖组合的质量,同时移除谱图中可能存在的Y离子,生成理论去糖峰谱图;(3)对所有去糖峰谱图,不需要设置任何*O*-糖基化的可变修饰,进行常规数据库检索,得到肽段序列的鉴定(见图4)。与传统设置*O*-糖基化可变修饰的检索方法相比,O-Search采用*O*-糖基化质量偏移检索策略,可以加快检索速度,提高86%的完整糖肽鉴定量,具有较高的灵敏度。同时,O-Search能够分析鉴定更多的*O*-糖结构,适合*O*-糖基化微观异质性的分析。

**图4:**
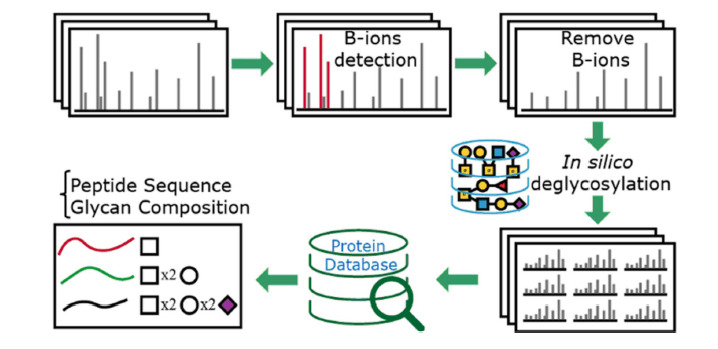
O-search检索策略用于解析完整*O*-糖肽HCD谱图^[37]^

MSFragger-Glyco是一个用于高灵敏度分析完整*N*-和*O*-糖肽的检索工具^[38]^,它使用了MSFragger开发的离子索引的开放修饰搜索方法和*O*-糖基化质量偏移检索策略,对文献*N*-和*O*-糖蛋白质组学数据进行再分析,能分别增加80%的注释N-糖肽谱图和一倍多的注释*O*-糖肽谱图。O-Pair Search是一种鉴定*O*-糖肽和*O*-糖基化位点的方法,它利用了基于碰撞解离和电子解离的质谱技术得到HCD-EThcD成对谱图,首先根据HCD谱图,通过离子索引的开放修饰检索鉴定完整*O*-糖肽;随后根据EThcD谱图,利用图论的方法进行修饰位点的定位^[39]^。

## 3 总结与展望

蛋白质糖基化作为一种重要的翻译后修饰,普遍发生在细胞膜蛋白和分泌蛋白中,在生物识别、信号转导和细胞通讯中发挥了重要的生物学功能。随着完整糖肽富集技术、质谱技术和谱图解析策略的革新,基于质谱的糖蛋白质组学成为分析位点特异性蛋白质糖基化的有力工具,并且使不同复杂生物体系中的糖蛋白质组学得到了越来越深入的研究。

用于富集完整糖肽的新材料基本都是基于现有的原理,优化材料的一些特性,比如亲水性、亲电性等,从而提高富集性能。考虑到糖蛋白质组学的临床应用,完整糖肽富集新技术更多地将富集方法整合进自动化的工作流程,以减少人工操作带来的误差、提高分析重现性和加大实验效率,使其更适应蛋白质糖基化相关的生物标志物的发现和疾病生理病理的研究等。质谱方法在近十年得到了飞速的发展,但适用于蛋白质糖基化的质谱方法依旧有很大的发展空间。除了本综述介绍的质谱碎裂方式以外,质谱数据采集模式,比如数据依赖采集(data dependent acquisition, DDA)、数据非依赖性采集(data independent acquisition, DIA)、MRM、平行反应监测技术(parallel reaction monitoring technology, PRM)等,在糖蛋白质组学中有很大的应用前景。相对应的,随着糖肽富集效率的提高和质谱方法的革新,谱图解析技术也得到了飞速的发展,其中在基于理论碎裂匹配策略或者谱图库匹配策略的完整糖肽鉴定方法中,数据分析的通量和假阳性的控制都非常关键。综上所述,糖蛋白质组学的新技术从富集方法、质谱方法、数据分析方法等层面都有很大的发展空间,这些也为糖蛋白质组学应用于临床疾病生物标志物的发现和蛋白质糖基化生物功能的探究等生命健康领域提供了强大的支持。
